# Successful treatment of a resistant invasive disseminated Fusarium infection in an immunocompetent patient

**DOI:** 10.1016/j.mmcr.2025.100750

**Published:** 2025-11-12

**Authors:** Frantzeska Frantzeskaki, Nikolas Stratopoulos, Sandra Elise van der Griend, Ioannis Renieris, Kostantinos Thomas, Iraklis Tsagkaris

**Affiliations:** a 2nd Department of Critical Care, Attikon University Hospital, National and Kapodistrian University of Athens, Medical School, Greece; b University of Leiden, Medical School, the Netherlands; c 4th Department of Internal Medicine, Attikon University Hospital, National and Kapodistrian University of Athens, Medical School, Greece

**Keywords:** Aspergillosis, *Fosmanogepix*, *Critically ill*, Immunocompetent

## Abstract

Fusariosis is a severe fungal infection associated with high mortality rates and an increasing incidence. However, its treatment remains challenging due to the frequent resistance of Fusarium species to conventional antifungal therapies. Here, we describe a case of an immunocompetent patient with invasive disseminated fusariosis with multifocal abscess formation after major traumatic injury. We report a significant clinical improvement with clinically relevant reduction of abscesses without major treatment toxicity after treatment with fosmanogepix.

## 1 Introduction

*Fusarium* species are ubiquitous environmental fungi commonly isolated from soil and water [1]. They are capable of causing both localized and disseminated invasive infections, particularly in immunocompromised individuals. The mortality rate associated with invasive fusariosis ranges from 60 % to 80 % [2,3], with persistent neutropenia representing the most critical risk factor [4], strongly associated with fatal outcomes.

The poor prognosis of fusariosis is, in part, due to the intrinsic resistance of many *Fusarium* species [5,6] to standard antifungal agents. Fosmanogepix is a novel antifungal prodrug that has demonstrated efficacy against *Fusarium* species in vitro [7,8] and exerts its antifungal activity by inhibiting Gwt1, an enzyme involved in the glycosylphosphatidylinositol (GPI) biosynthesis pathway. Inhibition of this pathway disrupts the anchoring of mannoproteins to the fungal cell wall and membrane, resulting in compromised cell wall integrity, impaired immune evasion, and attenuated virulence [9].

In this case report, we describe a 17-year-old immunocompetent patient diagnosed with invasive disseminated fusariosis characterized by multifocal abscess formation. The patient demonstrated significant clinical and radiologic improvement following administration of combination antifungal therapy that included fosmanogepix.

## 2 Case

A 17-year-old previously healthy male was admitted to a tertiary university hospital following a motorcycle accident. Upon arrival, the patient exhibited decreased level of consciousness and required endotracheal intubation. Whole-body computed tomography (CT) revealed severe cranioencephalic trauma, including multiple skull fractures, subdural hematomas, and subarachnoid hemorrhage. Additional injuries included an open fracture of the right shoulder and an open frontal scalp wound. An emergency craniectomy was performed, and the patient was transferred to the intensive care unit (ICU). Anti-edema therapy was initiated with intravenous mannitol and dexamethasone (8 mg three times daily for five days followed by gradual tapering). Due to escalating intracranial pressure and deteriorating cerebral edema, a barbiturate coma was induced using continuous intravenous thiopental (3 mg/kg/h) for ten days.

On day 18 of hospitalization (Day 0), the patient developed fever. Laboratory findings indicated a systemic inflammatory response, with elevated C-reactive protein (116 mg/L), procalcitonin (1.23 ng/mL), and leukocytosis (11,800 cells/mm^3^). Abdomen CT imaging demonstrated multiple renal abscesses (Fig. 1a). CT imaging of the right upper extremity showed subcutaneous air pockets consistent with necrotizing fasciitis. Blood cultures and swabs from the scalp wound grew *Fusarium* species, resistant to standard antifungal agents (Table 1) (Day 5). Identification of fungal isolates was based on their colonial characteristics and microscopic morphology. Moreover, sequenced based species identification was retrospectively performed. A column based method (QIamp^@^DNA mini Kit; Qiagen, Athens, Greece) that combined enzymatic and mechanical pre-treatment, was used for extracting genomic DNA from fresh subcultures grown on Sabouraud glucose agar supplemented with chloramphenicol and gentamycin. Polymerase chain reaction (PCR) followed by sequencing was used, while the internal transcribed spacer (ITS) region ITS1-5.8S-ITS2 and translation elongation factor 1α (TEF1α) were amplified for *Fusarium* isolates [10]. Antifungal susceptibility testing was performed in accordance with the European committee for antimicrobial susceptibility testing (EUCAST) broth microdilution (BMD) reference methodology [11].

**Figure:**
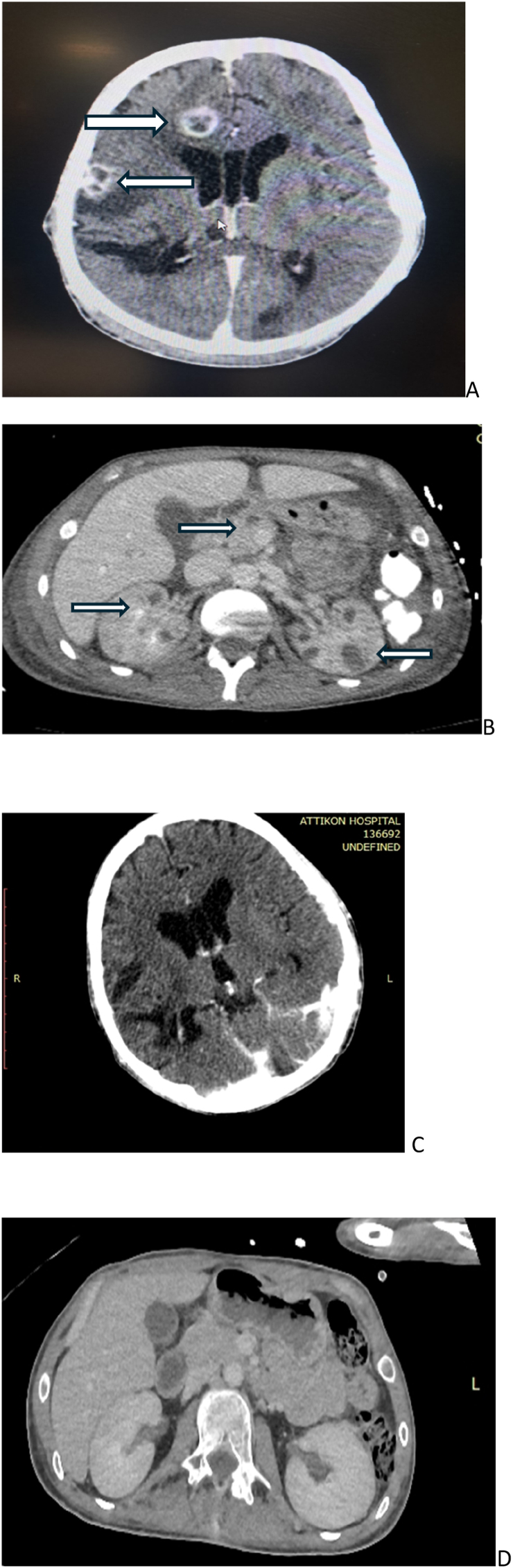
Radiological findings in of cerebrum and abdomen before and after fosmanogepix treatment. A) Abdominal CT imaging prior to antifungal treatment, showing bilateral kidney and pancreatic abscesses B) Brain CT before antifungal treatment, revealing multiple parenchymal hematomas and diffuse abscesses. C) Abdominal CT imaging after 5 months of treatment with fosmanogepix, revealing complete remission of kidney and pancreatic abscesses. D) Brain CT after 5 months of treatment with fosmanogepix, showing progressive improvement.

**Table 1:** Resistance profile expressed in Minimal Inhibitory Concentration (MIC) of the identified Fusarium species.

Voriconazole	MIC: 8 mg/L
Itraconazole	MIC: >8 mg/L
Posaconazole	MIC: >16 mg/L
Isavuconazole	MIC: >8 mg/L
Amphotericin B	MIC: 2 mg/L

A diagnosis of invasive disseminated fusariosis was established and the patient was screened for immunodeficiencies. HIV serology was negative, and neutrophils and immunoglobulin levels were within normal range, while the serologic tests for connective tissue diseases were negative. Brain MRI further revealed multiple parenchymal hematomas and diffuse microabscesses.

Targeted antifungal therapy was initiated with liposomal amphotericin B (7mg/kg, 450mg once daily), intravenous voriconazole (4mg/kg, 300mg twice daily) and anidulafungin (100mg once daily) (Day 5). Therapeutic drug monitoring of voriconazole was performed weekly and the trough levels ranged from 1.5 to 2mg/L. Surgical debridement of the infected scalp wound was also performed. Due to progression of the necrotizing soft tissue infection, right upper limb amputation was required. Three days after the initiation of antifungal therapy (Day 8), blood cultures became negative, inflammatory markers declined, and body temperature stabilized at 37.5 °C. Sedation was gradually weaned. However, the patient subsequently developed left-sided pupillary dilation (Day 12). Repeat brain CT revealed new enhancing intraparenchymal lesions and a midline shift (Fig. 1b). Concurrent abdominal CT demonstrated a hepatic lesion, multiple pancreatic lesions, and an increased number of renal abscesses. Additionally, cutaneous lesions appeared on the anterior thorax, abdomen, and right lower extremity. Direct microscopy of these skin lesions again identified *Fusarium* species. The patient was re-sedated, underwent tracheostomy, and antifungal therapy was continued.

Despite modest neurological improvement over the following three months (Day 113), repeat neuroimaging showed persistent cerebral and renal lesions. Based on consultation with infectious disease specialists, intravenous fosmanogepix was added to the antifungal regimen (600 mg once daily), and anidulafungin was discontinued. Fosmanogepix administration was initiated on ICU day 131, following emergency use authorization by the Greek Medicines Organization and written informed consent from the patient's legal guardians. In vitro susceptibility testing demonstrated high sensitivity of the isolated *Fusarium* strain to fosmanogepix. The drug was generally well tolerated, with nausea being the only notable adverse effect, managed by dividing the dose to 300 mg twice daily. Over the subsequent two months, the patient's neurological and motor function steadily improved. Follow-up brain and abdominal CT scans demonstrated significant regression of the previously identified lesions after four months of triple antifungal therapy with L-AMB, voriconazole, and fosmanogepix (Fig. 1c and d) (Day 233). A step-down in antifungal treatment was undertaken: L-AMB was discontinued, and the patient was transitioned to oral fosmanogepix.

During the period of his hospitalization, he developed several episodes of ventilator associated pneumonia, caused by *A. baumannii* and *K. pneumonia,* treated with combination of antibiotics, including ampicilline/sulbactame, meropenem, and ceftazidime/avibactam, according to susceptibility tests.

After a total of 10 months of hospitalization (Day 282), the patient was discharged to a rehabilitation facility on a maintenance regimen of oral voriconazole (200 mg twice daily) and fosmanogepix (400 mg twice daily). At a five-month follow-up, repeat brain CT confirmed complete resolution of enhancing lesions (Day 432). Clinically, the patient was alert, communicative, and breathing spontaneously without the need for tracheostomy. Antifungal therapy was discontinued after a total of 10 months of combined voriconazole and fosmanogepix administration (Day 533).

## 3 Discussion

Given the rising incidence of fusariosis and the high mortality associated with its disseminated forms, especially in immunocompromised populations, the development of novel anti-fungal agents is of paramount importance. In this report, we describe a rare case of invasive disseminated fusariosis in an immunocompetent adolescent, characterized by multifocal abscess formation and central nervous system (CNS) involvement. Disseminated *Fusarium* infections are most commonly reported in patients with prolonged neutropenia, hematological malignancies, or following hematopoietic stem cell transplantation [2,3]. However, in this case, the severity of the infection is likely attributable to a combination of extensive traumatic injuries with disruption of cutaneous barriers and the administration of high-dose corticosteroids for management of elevated intracranial pressure. These factors may have created a transiently immunosuppressed state, facilitating the widespread dissemination of *Fusarium*. As previously reported, *Fusarium* species exhibit a high level of intrinsic resistance to most conventional antifungal agents, including triazoles and echinocandins. Combination therapy with voriconazole and liposomal amphotericin B (L-AMB) has shown some benefit in case series and observational studies [12,13]. In pursuit of enhancing antifungal treatment, we decided to start triple combination therapy, based on in vitro data showing a positive immunopharmacologic effect of echinocandin on mold hyphae neutrophil killing, by the induction of β-glucan unmasking [14]. Nevertheless, the overall mortality of disseminated fusariosis remains high, particularly in patients with CNS involvement. Despite early initiation of dual antifungal therapy, our patient demonstrated refractory disease with the emergence of new abscesses in the brain, kidneys, pancreas, and cutaneous tissues. In light of the established antifungal resistance, fosmanogepix was introduced as part of salvage therapy. This resulted in substantial clinical and radiological improvement, and the drug was well tolerated, with only mild gastrointestinal side effects. The absence of neutropenia throughout the patient's ICU course and the lack of underlying chronic illness were likely contributory to the favorable outcome. Fosmanogepix (formerly APX001) is a novel, first-in-class antifungal prodrug that is metabolized to its active form, manogepix. While in vitro and in vivo preclinical data have demonstrated potent activity against *Fusarium* spp. [7,8], clinical experience with fosmanogepix remains limited. Few published cases describe its use in invasive, resistant fusariosis, and it has been deployed as compassionate therapy in an outbreak of healthcare-associated *Fusarium* meningitis in Mexico [15]. However, its efficacy and safety have not yet been fully established in larger, controlled clinical trials.

The optimal duration of therapy for fusariosis is not well defined and must be individualized based on clinical response and microbiological clearance. Winston et al. [16] reported a case of Fusarium infection of CNS in an immunocompromised patient, who improved after ten months of treatment with fosmanogepix. The antifungal was readministered three months after HSCT to prevent relapse. Goggin et al. [17] described a duration of treatment of approximately six months, following negative follow up skin biopsies, while Wu et al. [18] reported a four month treatment period. In our case, antifungal therapy was discontinued after ten months of combined treatment with fosmanogepix and voriconazole, following complete radiologic resolution of abscesses and sustained clinical improvement.

To our knowledge, this is among the first reported cases of disseminated fusariosis in an immunocompetent host successfully treated with fosmanogepix. The patient's clinical and radiologic improvement following the introduction of fosmanogepix, in conjunction with liposomal amphotericin B and voriconazole, suggests potential therapeutic benefit, particularly in infections resistant to standard treatments. Furthermore, the tolerability of fosmanogepix—aside from mild gastrointestinal side effects—supports its feasibility for long-term use. This case also highlights the importance of early identification, aggressive surgical debridement, and multidisciplinary management in the context of complex invasive fungal infections. The protracted clinical course and necessity for prolonged antifungal therapy emphasize the severity of disseminated fusariosis, even in immunocompetent individuals.

In conclusion, disseminated fusariosis is a rare but life-threatening infection that may occasionally occur in immunocompetent individuals in circumstances like traumatic injury and critical illness. This case underscores the potential role of fosmanogepix as an effective salvage agent in the management of invasive fusariosis refractory to standard antifungal regimens. Continued documentation of clinical experiences, along with data from ongoing trials, will be essential in defining the therapeutic role of fosmanogepix in invasive fungal infections. Collaborative data sharing and systematic case reporting remain critical to improving outcomes in this rare and challenging clinical entity.

## CRediT authorship contribution statement

**Frantzeska Frantzeskaki:** Writing – review & editing, Writing – original draft, Conceptualization. **Nikolas Stratopoulos:** Writing – original draft, Conceptualization. **Sandra Elise van der Griend:** Writing – original draft, Conceptualization. **Ioannis Renieris:** Writing – review & editing. **Kostantinos Thomas:** Writing – review & editing. **Iraklis Tsagkaris:** Writing – review & editing, Supervision, Conceptualization.

## Ethical form

We enclose the ethical form.

## Declaration of competing interest

There are none.
