# Supplementary material for: Successful treatment of a resistant invasive disseminated Fusarium infection in an immunocompetent patient
Source: Med Mycol Case Rep. 2025 Nov 12;50:100750. doi: 10.1016/j.mmcr.2025.100750 (PMC12663842; doi:10.1016/j.mmcr.2025.100750)
Supplement: Multimedia component 1 [file mmc1.docx]

Medical Mycology Case Reports
 ETHICAL FORM
 Article reference: MMCR_MMCR-D-25-00138


Medical Mycology Case Reports requires full disclosure of all sources of funding and potential conflicts
of interest. The journal also requires a declaration that the author(s) have obtained written and signed
consent to publish the case report from the patient or legal guardian(s).
If you have nothing to declare in any of these categories then this should be stated.

Funding Source

All sources of funding should be acknowledged and you should declare any extra funding you have
received for academic research of this work. If there are none state ‘there are none’.

Please state any sources of funding for your research
  **There are not any sources of funding**


Conflict o f Interest
Please declare any financial or personal interests that might be potentially viewed to influence the work
presented. Interests could include consultancies, honoraria, patent ownership or other. If there are none
state ‘there are none’.

Please state any competing interests

**There are none**


Consent
Please declare that you have obtained written and signed consent to publish the case report from the
patient or legal guardian(s).

Please state that consent has been obtained from the patient or legal guard ian(s)

**Written informed consent was obtained from the patient or legal guardian(s) for publication of this
case report and accompanying images. A copy of the written consent is available for review by the
Editor -in-Chief of this journal on request.**


As corresponding author, I hereby declare that I sign this document on behalf of all the authors
of the above mentioned manuscript.


Signature (a scanned signature is acceptable) Print nam e


 Frantzeska Frantzeskaki MMCR_MMCR-D-25-00138

_______________________________________ _____________ ______________ Manuscript number (if applicable) :
 Author name:
